# Driving Behavior Analysis of City Buses Based on Real-Time GNSS Traces and Road Information

**DOI:** 10.3390/s21030687

**Published:** 2021-01-20

**Authors:** Yuan Yang, Jingjie Yan, Jing Guo, Yujin Kuang, Mingyang Yin, Shiniu Wang, Caoyuan Ma

**Affiliations:** 1 Key Laboratory of Micro-Inertial Instrument and Advanced Navigation Technology, Ministry of Education, School of Instrument Science and Engineering, Southeast University, Nanjing 210096, China; 220203602@seu.edu.cn (Y.K.); 220203491@seu.edu.cn (M.Y.); 2 Jiangsu Provincial Key Laboratory of Image Processing and Image Communication, College of Telecommunications and Information Engineering, Nanjing University of Posts and Telecommunications, Nanjing 210003, China; yanjingjie@njupt.edu.cn; 3 School of Information Science and Electrical Engineering, ShanDong JiaoTong University, Jinan 250357, China; 18021018@stu.sdjtu.edu.cn; 4 School of Electrical and Power Engineering, China University of Mining and Technology, Xuzhou 221116, China; TS20130056A31@cumt.edu.cn (S.W.); Mcaoyuan@cumt.edu.cn (C.M.)

**Keywords:** driving behavior, trajectory analysis, pseudorange differential positioning, road information, BeiDou navigation satellite system

## Abstract

The driving behavior of bus drivers is related to the safety of all passengers and regulation of urban traffic. In order to analyze the relevant characteristics of speed and acceleration, accurate bus trajectories and patterns are essential for driver behavior analysis and development of effective intelligent public transportation. Exploiting real-time vehicle tracking, this paper develops a platform with vehicle-mounted terminals using differential global navigation satellite system (DGNSS) modules for driver behavior analysis. The DGNSS traces were used to derive the vehicle trajectories, which were then linked to road information to produce speed and acceleration matrices. Comprehensive field tests were undertaken on multiple bus routes in urban environments. The spatiotemporal results indicate that the platform can automatically and accurately extract the driving behavior characteristics. Furthermore, the platform’s visual function can be used to effectively monitor driving risks, such as speeding and fierce acceleration, in multiple bus routes. The details of the platform’s features are provided for intelligent transport system (ITS) design and applications.

## 1. Introduction

**Figure 1:**
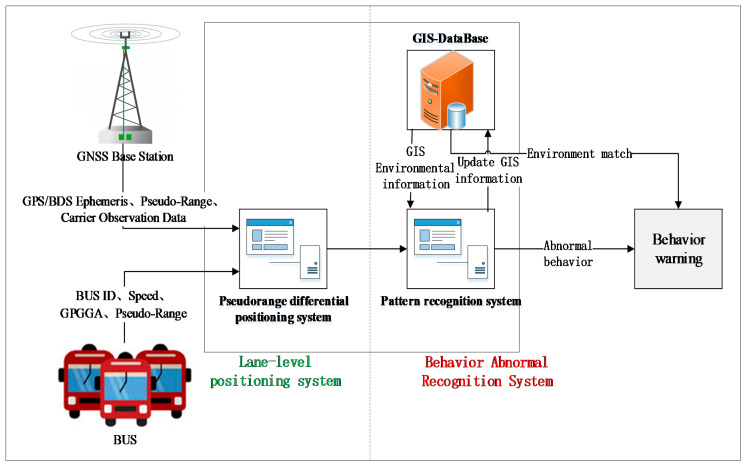
System structure of the proposed bus tracking and behavior analysis method.

Since the development of the first tram in Shanghai in 1908, public transportation in China has shown a history of more than 100 years. At present, the public transportation modes of urban residents in China mainly include bicycles, trams, buses, subways, and inter-city railways [1]. Because of low cost and high convenience, buses have become an indispensable means in urban public transportation services. In recent years, bus accidents have occurred frequently, threatening the lives of the masses and affecting the development of urban transportation. Bus drivers are closely related to achieving reliable and efficient travels, as the emergence of a series of distressing transport accidents and driving behavioral problems is becoming increasingly obvious [2]. Standardized traffic regulations and good driving behaviors are the guarantees for passengers to travel safely [3,4]. Currently, problems associated with driving behaviors for buses have become more prominent [5]. Risky driving phenomena, i.e., rapid starts and severe brakes (e.g., Figure 1), driving over the speed limits, frequent lane changes, shutdowns of positioning systems to avoid speed monitoring, and other undesirable phenomena, frequently occur [6,7]. As a result, complaints against bus drivers have sharply increased. The insufficient management and monitoring of buses have caused social issues, e.g., residents have gradually lost confidence in public transportation, social security systems and government programs. Consequently, a greater scientific basis and more statistical analyses are required to support intelligent transportation systems [8,9].

Bus traveling is the main mode of public transportation in most cities, with a large number of vehicles, pre-defined routes, a wide travel range, and high passenger capacity. They travel on fixed routes daily spread all over cities, which are more representative than other vehicles and have a significant impact on traffic infrastructure constructions. For bus transportation systems, multiple factors affect passenger comfort, travel safety, and traffic performance. Bus drivers are an essential factor that affects the safety of urban public transportation. Their driving behaviors, especially bad driving behaviors, are the main issue causing traffic accidents and disorders, e.g., a large number of accident cases show that human activities are crucial in traffic accidents [10,11]. As such, the driving behaviors of bus drivers are necessary to be studied, as well as their driving characteristics, to improve the safety management of and reduce the traffic accidents in public transportation. By providing statistical data related to the modes of bus lines and enabling coordinated modes for various users, to be better informed, will lead to a safer, more efficient, and smarter bus network.

Recently, driving behaviors had been studied by scholars through different techniques [12]. Academics aim to estimate the bus mobility (i.e., speed and acceleration) [13], productivity (i.e., passenger capacity and coverage) [14], performance (i.e., comforts and waiting time), and safety (i.e., potential accidents and traffic violations) for passengers, traffic planners, and road users. Firstly, bus driving estimation relies on the ability to precisely estimate vehicle speed and acceleration. There are various techniques to acquire continuously generated bus traffic information [15]. Traditional approaches use wheel odometers or loop detectors to detect vehicle movements, but the main drawback is that they are easily affected by vehicle conditions and environments. Video-based road traffic monitoring and prediction have advantageous in terms of flexibility and human visualization. However, its computational complexity is high [16]; for instance, an internal camera that captures the face of the driver [17] or an external camera that captures images of the road [18]. A global navigation satellite system (GNSS) is the most promising positioning and tracking technique, including differential GNSS, GNSS precise point positioning, and real-time kinematic GNSS [19]. The 2D/3D range detection (LiDAR) [20] and millimeter-wave radar (MWR) [21] methods are also used to monitor the environment. With continuous position tracking and road information, the vehicle speed estimation, environment identifications, and traffic prediction provide references for the study of the driving safety for city buses [22].

Bus monitoring systems needs real-time operation, insensitivity to environments, and low complexity [23]. GNSS traffic estimation allows for real-time, efficient, wide-region, and inexpensive use of a stand-alone device [24]. However, GNSS errors are caused by multipath and NLOS (non-line-of-sight) effects, which become severe in urban areas [25]. In particular, GNSS pseudorange positioning is not accurate enough for lane-level bus tracking [26]. Differential GNSS (DGNSS) [27] explores a fixed station with known coordinates and a moving receiver with unknown coordinates [28]. The fixed station, and the moving receiver that observes the same satellite conditions, can minimize the multipath errors based on the error correction based on the fixed station. Furthermore, a class of positioning approaches combined GNSS with the aid of an MEMS inertial measurement unit (MIMU of acceleration sensors, gyro sensors, and compass) [29], road network [30], and global system for mobile communication (GSM) network [31]. Li et al. proposed a roadside equipment integration method to integrate the received signal strength (RSS) and GPS [32], while GNSS always observed the transmission errors from the hardware and environments of the receiver from satellites to the surface of the earth [33]. In particular, GNSS in urban areas obtained noisy distance measurements due to severe multipath signals, which made it difficult to achieve lane-level position estimations, with a precision of only 10 m to 50 m [34]. The literature [35] has explored global positioning systems (GPSs) for lane-level positioning, which achieved a higher positioning accuracy and low complexity. The driving trajectory, speed, and acceleration values of a bus driven by its corresponding driver were intuitively expressed by the precise positioning of a BeiDou navigation satellite system (BDS); e.g., Wei et al. in [15] introduced a decentralized vehicle remote positioning based on multiple available navigation satellite systems and mobile networks [36]. The considerable challenges of GNSS hinder accurately estimating the urban-wide bus trajectory and speed, including (a) the pseudorange errors sourced from the urban multipath environments [37]; (b) missing data of the GNSS positioning in non-line-of-sight urban scenarios [38]; (c) low-frequency sampling and estimations from the GNSS measurements; and (d) real-time estimations of multiple bus lines over urban-wide areas.

Bus vehicle tracking methods focus mainly on algorithms and technologies; however, the application domain is seldom summarized. In this paper, we provide a review of bus vehicle tracking approaches in urban environments. This study investigates real-time positioning data acquired from GPS/BDS differential modules installed in the test vehicles, and the behaviors of multiple bus lines are analyzed. The proposed bus trajectory, speed, and acceleration estimations by its corresponding driver can be intuitively expressed by the lane-level positioning of the DGNSS. Besides, it is coupled with road information, which facilitates the identification and analysis of driver patterns. The bus driving data simultaneously estimated by the DGNSS traces can be applied to many other applications for intelligent transportation systems and bus traffic priorities; for instance, an intelligent traffic-light control needs a bus traffic heat-map. Overall, this work provides valuable information about the public traffic on a city-wide scale in China and can help better evaluate passengers, bus company managers, and government planners. The rest of this paper is organized as follows. Section 2 describes the system structure and data acquisition of the real-time DGNSS traces for multiple bus lines. In Section 3, we design and establish the trajectory and motion estimation methods. Section 4 verifies the analysis of the proposed processing by Python of the bus traffic data in city-wide field tests. Section 5 concludes this work with a discussion of future research.

## 2. Bus DGNSS Tracking and Behavior Analysis System

This study presents a pseudorange differential positioning based on GPS/BDS fusion to enhance city bus tracking. In parallel, it also introduces a framework to estimate bus driving with velocity and acceleration. Furthermore, the verification of this work is the empirical estimation of multiple bus networks in urban areas within one week in China. A statistical analysis is proposed that yields risky driving behaviors and concludes the correlation between driving behaviors and traffic conditions.

### 2.1. System Structure

A bus is an indispensable means of transportation for urban residents. The supervision and control of bus traffic have long been a concern for various users, i.e., city residents, bus companies, and governments, etc. Bus traveling observes a wide variation in terms of vehicle delays, vehicle speed and travel time, travel routes, traffic signal controls, dynamic traffic flows, and operating characteristics. Bus tracking and behavior analysis are of great significance to improve public traffic capability. Consequently, a positioning and analysis system is a vital foundation to ensure the safe operation of buses.

We established a real-time DGNSS tracking function for buses for the field tests in Nanjing, China, with the lane-level positioning accuracy based on a vehicle-mounted terminal. The processing of bus data mainly completes the management and analysis based on the GPS/DBS data, which elaborates the status of the buses, vehicle trajectories, individual driving characteristics, and road environments. The proposed system extracts vehicle information from the bus monitoring in two parts: the bus tracking subsystem and the behavior analysis subsystem; the structure of the system is illustrated in Figure 1. The first subsystem (the left part of Figure 1) is composed of a real-time GPS/DBS acquisition terminal (installed in the bus vehicles) and a background pseudorange differential position estimation; the second subsystem includes the behavior analysis and warnings (the right part of Figure 1), incorporating the database of the trajectory estimations, vehicle information, road information, and GIS (Geographic Information System). Overall, the system converts the DGNSS data into bus spatiotemporal information that can be analyzed; then, the driving behavior analysis is conducted to ensure the safety of the buses.

The main information collected by the bus positioning terminal includes time, bus number, bus ID, and real-time DGNSS data. Among them, the reliability of the positioning information is the most important to dynamically and continuously monitor the real-time location and travel status of buses. The background GNSS pseudorange differential estimation [35] can be further divided into three units according to its function: a vehicle terminal monitoring unit, a CORS base station connection unit, and a pseudorange differential unit [39].

### 2.2. Differential DGNSS Pseudorange Traces

Lane-level positioning technology is the basis for achieving smart transportation, autonomous driving, and other fields, especially in the monitoring of the behavior of drivers for special vehicles such as buses. According to the relevant standards, the width of each road lane is 3.5 m in the city, 2.3–2.5 m at intersections, and 3.75 m on main roads (including expressways). Therefore, the positioning error of a bus should be within ±2.3 m; that is, a submeter positioning of a bus can ensure lane-level positioning. The real-time information collection of buses is responsible for collecting the bus information, the differential optimization of their location, and map information.

Our system ensures the lane-level positioning requirements of buses based on a real-time GNSS differential positioning module. The system ensures the submeter-level estimations of buses and provides data support for further driving behavior analysis. We used network pseudorange differential positioning, with a rover located in the middle of multiple base station networks and multiple base station pseudorange corrections (PRCs) for interpolating to obtain a comprehensive PRC for the differential positioning. The workflow is as follows:

First, collecting the pseudorange observations of all base stations in the base station network through the Internet; next, the data processing center preprocesses the data, and calculates the PRC and range rate correction (RRC) of all the reference stations corresponding to each satellite through the precise coordinates of the reference station; and then, according to the user coordinates requesting the service and the interpolation model, the integrated PRC of the user of the rover is obtained; finally, the generated integrated PRC and RRC are RTCM encoded and sent to the rover. After the rover corrects its pseudorange observations, the differential positioning is realized. When there is a base station close enough to the rover user, the current closest base station correction number is used as the comprehensive PRC and sent to the user; when the rover station is far from all base stations, in this paper, the inverse distance weight interpolation model is used to generate the PRC of the rover. The interpolation correction number model is mainly based on triangles as the solution unit, and the model needs to meet the following conditions.

(1) $$\left\{ \begin{array}{l} d_{i}=\sqrt{{\left(X-x_{i}\right)}^{2}+{\left(Y-y_{i}\right)}^{2}+{\left(Z-z_{i}\right)}^{2}}\\ s_{i}=1/d_{i}\\ s=\sum_{i=1}^{3}s_{i}\\ q_{i}=s_{i}/s \end{array}\right.$$

In the formula, $d_{i}$ represents the distance between the reference station and the rover, and $q_{i}$ is the final weight; then, the final integrated PRC of the rover is

(2) $$PRC_{R}(t)=q_{1}\times PRC_{1}(t)+q_{2}\times PRC_{2}(t)+q_{3}\times PRC_{3}(t)$$

It can be seen from the calculation conditions of Equation (6) that q1+q2+q3=1. For the same satellite, each reference station obtains the same satellite clock error, so the integrated error correction number satellite clock error remains unchanged, which is the same as the satellite clock error calculated by the rover through the satellite ephemeris; thus, the influence of the satellite clock error can be eliminated.

The application process of the pseudorange differential positioning based on BeiDou/GPS fusion is similar to that of the network RTD differential positioning. Because of the difference in accuracy between the BeiDou and GPS systems, different weights are used for the two systems to avoid the BeiDou system from reducing the differential positioning accuracy when the GPS satellites are of good quality. The dual-system fusion pseudorange differential positioning model is as follows.

Assuming that the coordinates of the rover *R* are (*X, Y, Z*), and the current epoch can observe *r*1 BDS satellites and *r*2 GPS satellites, then the pseudorange observation equations of the *m*-th (1 ≤ *m* ≤ *r*1) BDS satellite and the *n*-th (1 ≤ *n* ≤ *r*2) GPS satellite from the *i*-th base station are

(3) $$\begin{array}{l} \rho_{BA_{i}}^{m}=R_{BA_{i}}^{m}+c\delta t_{BA_{i}}-c\delta t_{B}^{m}+I_{BA_{i}}^{m}+T_{BA_{i}}^{m}+\varepsilon_{\rho }\\ \rho_{GA_{i}}^{m}=R_{GA_{i}}^{m}+c\delta t_{GA_{i}}-c\delta t_{G}^{m}+I_{GA_{i}}^{m}+T_{GA_{i}}^{m}+\varepsilon_{\rho } \end{array}$$

Among them, $\rho_{BA_{i}}^{m}$ and $\rho_{GA_{i}}^{m}$ are the pseudorange observation values of base station A to the *i*-th BeiDou and GPS satellites, respectively; $R_{BA_{i}}^{m}$, $R_{GA_{i}}^{m}$ are the distance between the *i*-th satellite and base station A; $c\delta t_{BA_{i}}$/$c\delta t_{GA_{i}}$ and $c\delta t_{B}^{m}$/$c\delta t_{G}^{m}$ are the clock error of the reference station A receiver and the satellite clock, respectively; $I_{BA_{i}}^{m}$/$I_{GA_{i}}^{m}$ and $T_{BA_{i}}^{m}$/$T_{GA_{i}}^{m}$ are the ionospheric and tropospheric delay errors; and $\varepsilon_{\rho }$ is the pseudorange noise.

According to the calculation method of the network RTD correction number, calculate the current rover’s comprehensive PRC for the BDS satellite and GPS satellite, respectively. Use the synthetic PRC $\sum_{i=1}^{3}PRC_{BR_{1}}(t)$, $\sum_{i=1}^{3}PRC_{GR_{1}}(t)$ obtained by the rover R to correct the observed values $\rho_{BA_{i}}^{m}$ and $\rho_{GA_{i}}^{n}$ of the rover as follows:

(4) $$\begin{array}{l} \rho_{BA_{i}}^{m}+\sum_{i=1}^{3}PRC_{BR_{1}}(t)=I_{BR}^{m}-\sum_{i=1}^{3}I_{BA_{i}}^{m}+T_{BR}^{m}-\sum_{i=1}^{3}T_{BA_{i}}^{m}\\ \rho_{GA_{i}}^{n}+\sum_{i=1}^{3}PRC_{GR_{1}}(t)=I_{GR}^{n}-\sum_{i=1}^{3}I_{GA_{i}}^{n}+T_{GR}^{n}-\sum_{i=1}^{3}T_{GA_{i}}^{n} \end{array}$$

Use the corrected pseudorange observation values to construct an observation equation, which gives different weights to the BDS and GPS, increasing the weight value when there are more satellites in the GPS. Then solve the precise coordinates of the rover.

In our field tests, the positioning performance of the GPS/BDS combined system is better than that of a GPS or BDS single system. Therefore, the proposed vehicle terminal adopts a GPS/BDS dual-mode positioning, with the relevant parameters listed in Table 1.

**Figure 2:**
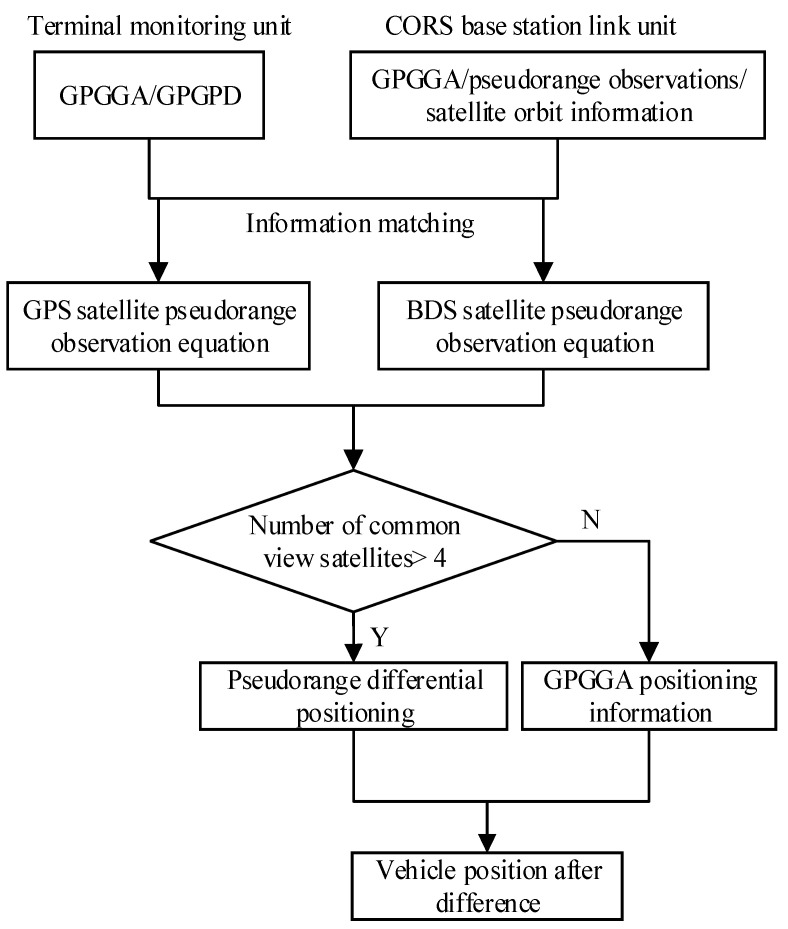
Flow chart of the GPS/BDS pseudorange differential method.

Other studies have discussed the algorithm of the pseudorange differential relative positioning in detail [40]. The information of the buses includes the on-board terminal number and submeter position estimation, and speed constitutes the real-time information of the buses. The flow of the pseudorange differential unit (Figure 2) is described as follows:

(1) The vehicle’s GPGGA and GPGPD parameters of the DGNSS obtained by the on-board terminal monitoring unit are matched with the pseudorange observations and satellite orbit information by the CORS base station connection unit.
(2) The pseudorange observation equations of the GPS and BDS data are generated separately.
(3) If the number of available satellites is greater than 4, the pseudorange differential positioning is performed; otherwise, the GPGGA positioning data are used.
(4) According to (3), the submeter position estimations can be obtained when the residual error and difference analysis are determined.

The driving behavior analysis, integrating the road information, is one of the important extensions for bus management. It is mainly composed of a road network database, behavior analysis methods, and an abnormal behavior alarm module. The behavior analysis module explores the trajectory data of the vehicle by the lane-level DGNSS positioning. It also analyzes the driving statuses of the vehicle according to the position trajectory, speed, acceleration, and other information to warn of any abnormalities. If an abnormal situation occurs, the corresponding road section in the road network server [41] is referred to to examine the abnormal situation, simultaneously categorizing whether the driver has violations and other improper behaviors, which reduces the risk of accidents [19]. The system can also provide support for the analysis of the cause of an accident offline. For further improvements of the positioning accuracy and reliability, one can incorporate the GNSS integrity [42] and space diversity [43] in urban areas.

### 2.3. DGNSS-Based Bus Road Networks

Road networks can be generated by digitization maps, mobile survey vehicles, or aerial photographs and GNSS trajectories [44]. Lane-based road networks are essential for bus route planning, such as lane locations and lane changes. Our system implements a lane-level road network from the DGNSS estimations of the bus vehicles with the following procedure and results.

(1) DGNSS trajectories are gathered from multiple travel rounds of an individual bus route, in the daytime and night real-world tracking, respectively.
(2) A Kalman filter is adopted for trajectory optimization to avoid the severe vibration GNSS multipath and NLOS errors.
(3) The refined trajectory is associated with road sections according to the corresponding bus vehicle identification (ID), as the basic unit for lane information extraction.

**Figure 3:**
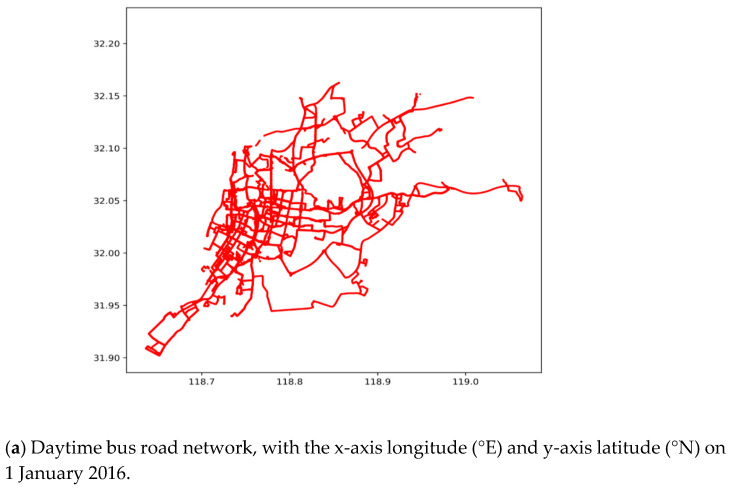

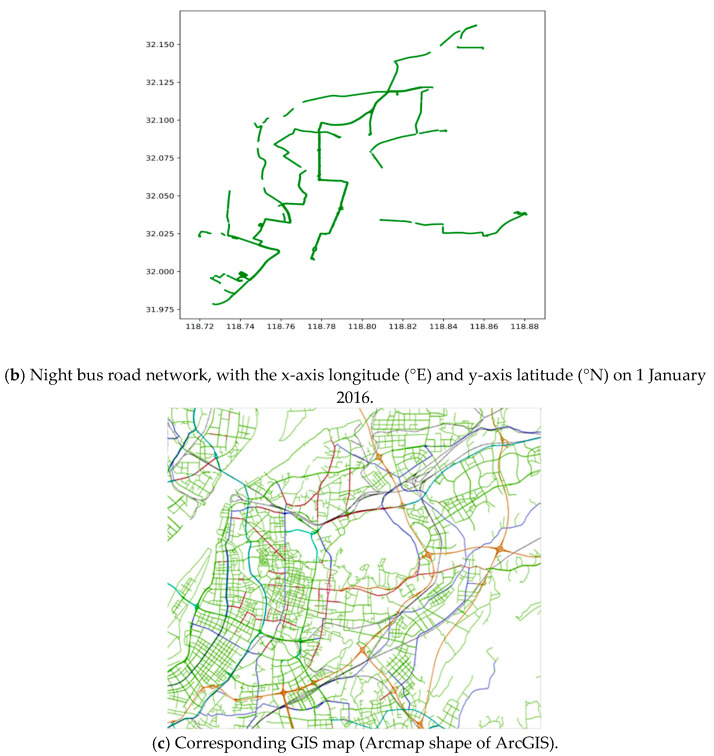
Bus road network and GIS map in the urban area of Nanjing city in China, according to the scatter plot of all DGNSS tracking points on 1 January 2016.

Figure 3 illustrates the bus road network, with the daytime and night DGNSS traces in the urban area of Nanjing city in China.

Figure 3 illustrates that the DGNSS records can automatically achieve the lane-level trajectory tracking and detailed bus road networks. Especially, the bus road map in Figure 3a is representative of the city road map in Figure 3c, indicating that the daytime bus of Nanjing city almost covers the whole urban area. Figure 3a,b display the daytime and night bus road network by the DGNSS estimations, respectively, which describe the difference between the daytime and night bus routes. Furthermore, the data of each lane, including the directions and intersections, can be extracted by real-time tracking. Nevertheless, the bus road network lacks the driving characteristics. Consequently, the speed and acceleration characteristics need to be extracted from the DGNSS traces, and the driving behaviors are estimated based on data mining of the road information.

## 3. Estimations and Characteristics of Bus Driving

### 3.1. Estimation of Bus Driving Data

**Figure 4:**
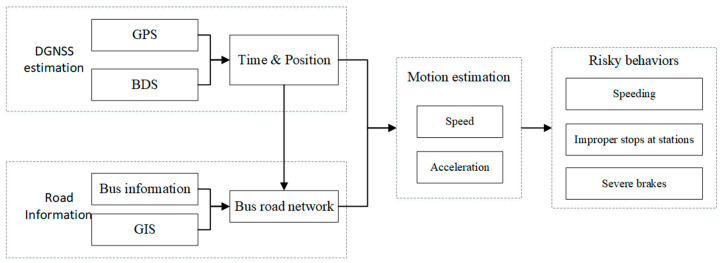
Flow chart of the bus driving statistics and behavior analysis.

Buses driving characteristics and behaviors are crucial for ensuring reliable and safe travels. Various elements can be used to evaluate bus driving behaviors, i.e., speeding, delay time, and improper stopping [45]. We investigated the evaluation of speeding, severe braking, and stop quality at the bus stations, as in the flow chart below, depicting the analysis of driver behavior (Figure 4).

The following statistics can be estimated for the bus driving evaluations.

- Speeding: Different roads of urban traffic has different traffic speed limits [5]. Speeding is not only a traffic violation but also a danger to passengers’ safety. In this study, the speed limit information of the local road traffic is set in the database, and the bus speed is estimated by the filtered DGNSS positioning data. Therefore, the bus speed can determine whether the bus is speeding.
- Rapid starting and braking: A typical characteristic is that the acceleration of a vehicle is greater than a certain threshold. A driver’s excessive acceleration degrades passages’ comfort and safety. The thresholds of “Rapid starting and braking” were based on acceleration as referred to in [46].
- Stopping at bus stations: Bus drivers often avoid to stop at some designated stations or stops out of station areas. As such, this study considers whether buses accurately stop at the corresponding stations. The typical feature of vehicle stopping is a vehicle speed of 0 lasting over 10 s.
- Average one-way duration: This parameter is used as the bus route’s overall driving evaluation [47].
- Fatigue driving: Bus traffic rule defines that fatigue driving is more than 4 h of continuous driving. Fatigue driving may probably lead to traffic accidents [48].

A vehicle-mounted DGNSS receiver can obtain information such as current locations in real-time. To reduce the impact of abnormal data on driving behavior analysis, the GNSS track data needs to be cleaned and estimated as follows.

(1) Speed estimation

For the statistical analysis, the variables involved are defined as follows: T is the time, V is the speed, W is the latitude, J is the longitude, H is the direction angle, and ID is the driver’s identification number. Then, the information of each sampling sequence of the DGNSS data is I={T,V,W,J,H}, and the variables of each sampling sequence k are recorded as $I_{k}=\{T_{k},V_{k},W_{k},J_{k},H_{k}\}$.

The vehicle speed information can be obtained from the vehicle’s GNSS trajectory data. Set the positioning coordinate of any past GNSS point Pi and the new GNSS point is Pj, with the sampling sequence i and j. One can calculate the time interval by j-i, since the sampling frequency is 1 Hz. And the average speed of the time interval (i,j) is calculated by the Euclidean distance (‖ ‖) of the two coordinates as

(5) $$V_{\mathrm{avg}}=\frac{\left\|Pj-Pi\right\|}{j-i}$$

For the sampling sequence k, the speed is

(6) $$V_{k}=\frac{\left\|P_{k}-P_{k-1}\right\|}{\left(T_{k}-T_{k-1}\right)}=P_{k}-P_{k-1}$$

The data acquisition frequency of the GNSS receiver is 1 Hz, and the maximum speed ($V^{\max}$) of the road section is set to 60 km/h (in other words, 16.67 m/s). Thus, the maximum distance between two adjacent GNSS points should be smaller than 16.67 m, which can be used as the threshold value to detect and correct the abnormal tracking data. The setting of the speed threshold has a decisive influence on the determination of speeding behaviors.

(2) Rapid acceleration and deceleration estimation

According to the calculated speed, the acceleration or deceleration $A_{k}$ between two adjacent GNSS track points at the sampling sequence k can be estimated.

(7) $$A_{k}=\frac{\left(V_{k}-V_{k-1}\right)}{\left(T_{k}-T_{k-1}\right)}=V_{k}-V_{k-1}$$

Set the rapid acceleration threshold as $A^{\mathrm{acc}}$, and the rapid deceleration threshold as $A^{\mathrm{dec}}$, which can be used to determine a sudden motion.

### 3.2. Characteristics of the Bus Driving Data

When the above estimations indicate driving risks, such as speeding, rapid acceleration, and rapid deceleration, the number of dangerous behaviors, duration, accumulated distance, and road sections can be calculated on the basis of subjective empirical values, with the acceleration threshold $A^{\mathrm{acc}}$ for the rapid acceleration and the acceleration threshold $A^{\mathrm{dec}}$ for the rapid deceleration. The relevant variable definitions of the characteristics of various driving behaviors are shown in Table 2.

When a vehicle is speeding, the distance, duration, and the number of speeding events are accumulated, as shown in Equations (8)–(10). In the same way, the cumulative distance, duration, and number of sharp shifts are calculated according to Equations (11)–(16).

(8) $$VS=VS+\Delta S,V_{K}>V^{*}$$

(9) $$VT=VT+T_{K}-T_{K-1},V_{K}>V^{*}$$

(10) $$VN=VN+1,V_{K}>V^{*}\;\&\;V_{K-1}<V^{*}$$

(11) $$AAS=AAS+\Delta S,A_{K}>A^{*}$$

(12) $$AAT=AAT+T_{K}-T_{K-1},A_{K}>A^{*}$$

(13) $$AAN=AAN+1,A_{K-1}<A^{*}\;\&\;A_{K}>A^{*}$$

(14) $$ASS=ASS+\Delta S,A_{K}<A\prime$$

(15) $$AST=AST+T_{K}-T_{K-1},A_{K}<A\prime$$

(16) $$ASN=ASN+1,\;A_{K}<A\prime \;\&\;A_{K-1}>A\prime$$

Larger values of VS,VT,AAS,AAT,AST,ASS indicate the higher duration and percentages of speeding, as well as severe accelerations; that is, the driving behavior is dangerous. Otherwise, the phenomenon is a coincidence rather than a risky behavior. Overall, the characteristics of the driver’s dangerous driving behavior in a certain duration can be evaluated.

## 4. Field Tests and Behavior Analysis

### 4.1. Implementation of Real-Time Bus Tracking

The bus real-time tracking system is divided into two parts: the DGNSS terminal and background realization. The hardware is mainly the installation of the vehicle terminal and the establishment of the background server. The realization of the software mainly depends on the development of the background pseudorange differential estimations.

(1) Terminal hardware and server platform

**Figure 5:**
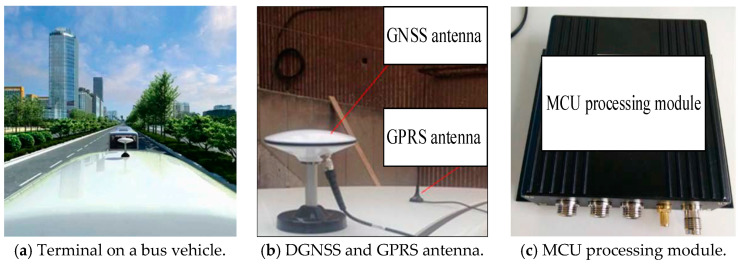
Hardware of the DGNSS terminal on a bus vehicle.

We designed a vehicle-mounted terminal and a background server platform to process the bus driving data, as shown in Figure 5. To implement the real-time applications, the proposed terminal integrates additional components such as a transceiver for 3G communication and serial ports on a development board. The software realization depends on the GNSS pseudorange differential module. In our study, the bus on-board terminal is installed outside the vehicle, as shown in Figure 5a; it contains the DGNSS antenna and GPRS antenna, as shown in Figure 5b; the other part, namely, the MCU processing module, is installed in the vehicle, as shown in Figure 5c.

(2) GNSS pseudorange differential module

The back-end pseudorange differential module was designed based on the MFC environment and adopts the Microsoft Studio 2010 (C++) environment. We divided the software visual interface into 4 groups: (1) System: the monitoring server CPU, memory and communication, etc.; (2) terminal service: it realizes the function of a terminal monitoring unit; (3) CORS: it realizes the function of base station connections; and (4) parameters: it configures the communication parameters, for instance, the terminal port number between the server and the terminal.

The back-end pseudorange difference server obtains observations at each time sequence and estimation the current position, time, and number of satellites. The returned data format is shown in Table 3.

The observations, estimations, and prior knowledge (i.e., bus information and road information) are stored in .dmp format and exported to the PL/SQL Developer by Oracle database.

### 4.2. Bus Driving Behavior Analysis

This paper studies the bus tracking statistics and behaviors of some urban bus routes in Nanjing, China. For privacy and security, the data were cleaned in the data preparation stage (e.g., we use Bus No. xxx instead of a particular bus route). In the modeling stage, we applied the deep learning library “turiCreate” to correlate the bus route in Python, i.e., the GNSS signal, speed, acceleration, road sections, bus route, etc. Based on the data visualization, the characteristics of bus traffic were figured out.

**Figure 6:**
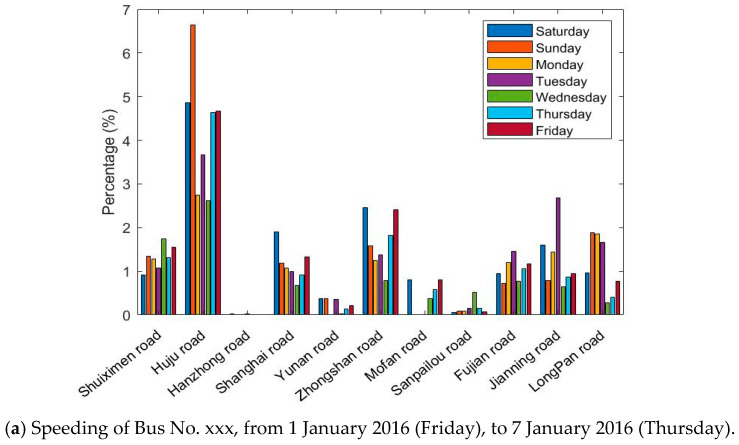

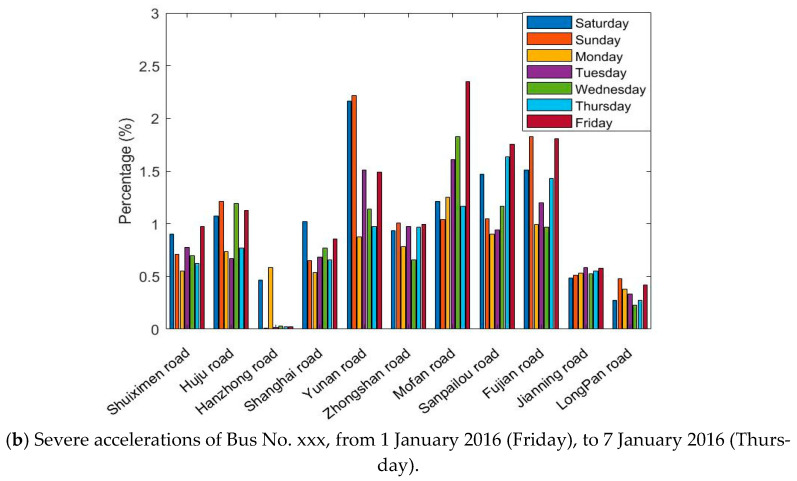
Percentage of speeding and severe accelerations for the whole data instances, for the Bus No. xxx line on 11 roads in 7 days.

The GPS/BDS data were processed and filtered to eliminate abnormal values that do not fit the actual situation. Statistical results of the DGNSS traces of Bus No. xxx in one week (from 1 January 2016 (Friday), to 7 January 2016 (Thursday)) were investigated (Figure 6).

Figure 6 shows the situation of exceeding the standard speed and acceleration on 11 roads in 7 days. The absolute number of speeding or accelerations is not comparable on each road; therefore, we sorted the percentage of the speed and acceleration durations compared with the total time spent in each road section. Although the bar graphs greatly differ, one can observe that

- bus driving in Huju Road always results in much higher percentages of speeding than the other roads, but fewer accelerations;
- no speeding is recorded in Hanzhong Road for 7 days, and only a few high accelerations were present;
- the percentages of speeding and severe accelerations are highly dependent on the road sections;
- the driving behaviors also vary on different days, which is hard to summarize.

According to the road information, Huju Road has a speed limit of 40 km/h, and the nearby road limits are 50 km/h. Hanzhong road only two narrow lanes with a speed limit of 50 km/h, many traffic lights, and surrounding hospitals and schools. Speeding and severe accelerations occur frequently on the whole routes during the week. The drivers are more likely to drive according to road and traffic conditions and tend to make sudden stops.

**Figure 7:**
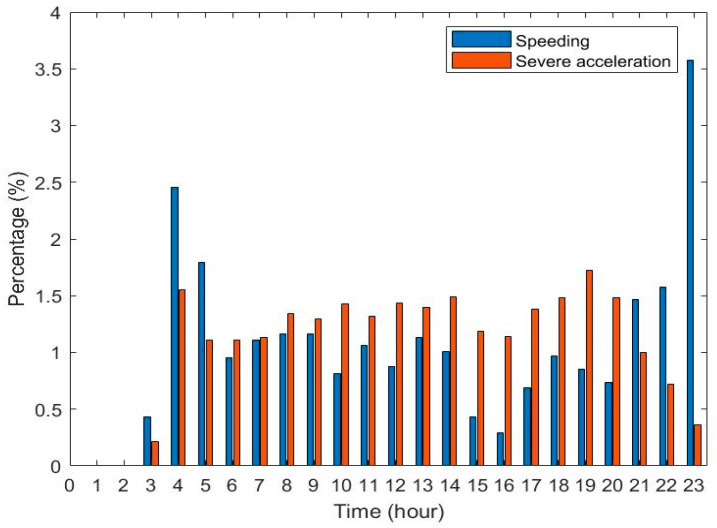
Bus No. xxx speeding and severe accelerations in 24 h of 7 days.

Figure 7 shows the percentage of speeding within 24 h within 7 days. The speeding of the first bus in the early morning and the last bus in the late night are very high. A much lower percentage of speeding is observed at 3, 15, and 16 o’clock; the driver of Bus No. xxx often leads to speeding in the early mornings and late nights when traffic is low. The speeding is lower in the afternoon when drivers’ fatigue and the traffic flow are higher. Compared with speeding, high accelerations or sudden breaks do not correlate with time.

**Figure 8:**
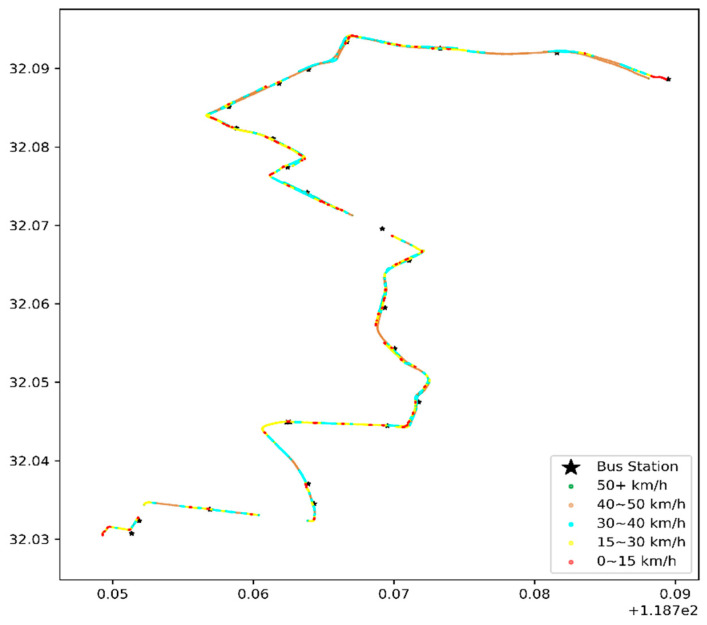
Heat map of the vehicle speed of the first bus in the early morning, with the coordinates of the x-axis longitude (°E) and latitude (°N) on 1 January 2016.

**Figure 9:**
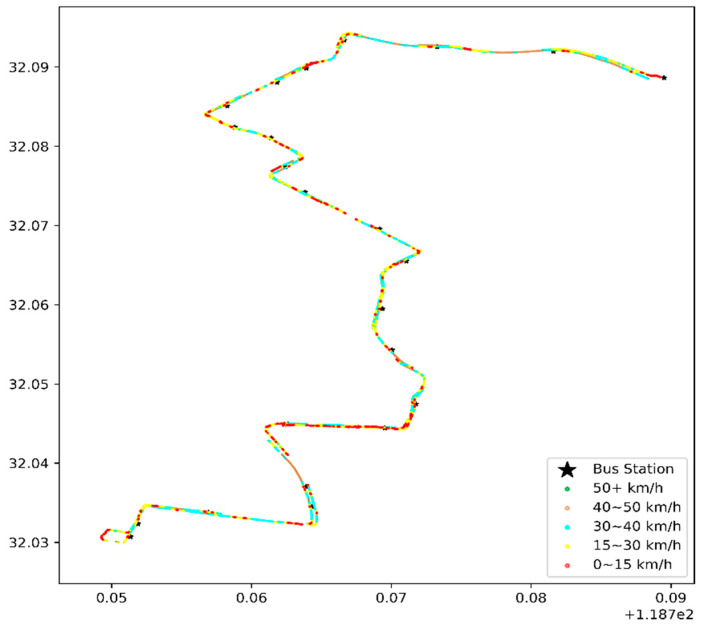
Heat map of the vehicle speed during morning rush hours, with the coordinates of the x-axis longitude (°E) and latitude (°N) on 1 January 2016.

The data on 1 January 2016, were selected for a one-day heat-map analysis, as in Figure 8 and Figure 9.

(1) Congestion or speeding

Figure 8 and Figure 9 illustrate the bus travel quality in terms of displaying detailed trajectories, and the red dots (the slow speed 0–15 km/h) are congestion conditions in the corresponding road sections. The morning rush hour bus suffers more congestion than the first bus in the morning, especially on Hanzhong Road.

**Figure 10:**
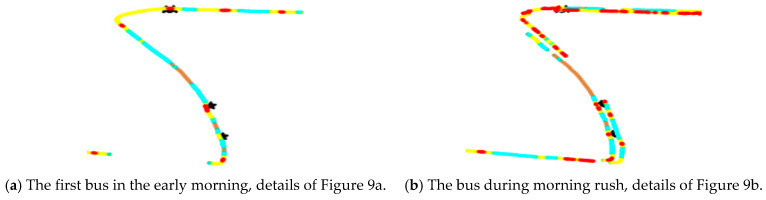
Detailed heat scatter plot of the vehicle speed in the section on Huju Road, from Figure 9.

The difference between Figure 10a,b shows that the speeding (green point) of the first bus is higher, which is consistent with the previous analysis. As shown in Figure 10a, the disconnected parts of the scatter plot indicate that Huju Road has many GPS outage conditions. Before the data are lost, the vehicle speed exceeds 50 km/h. After the data are recovered, the speed is still 50 km/h. According to our field interview, some drivers turn off the GPS/BeiDou positioning module during data measurement to avoid speeding fines, which results in losing some positioning data.

(2) Emergency brake/stop

The number of accelerations that exceeds the threshold value was analyzed. A driver completed 7 round trips in one working day, averaging 18 times of severe stopping/accelerations per trip. Some data instances are presented in Table 4, which shows the acceleration.

(3) Whether the bus stop properly at bus stations

**Figure 11:**
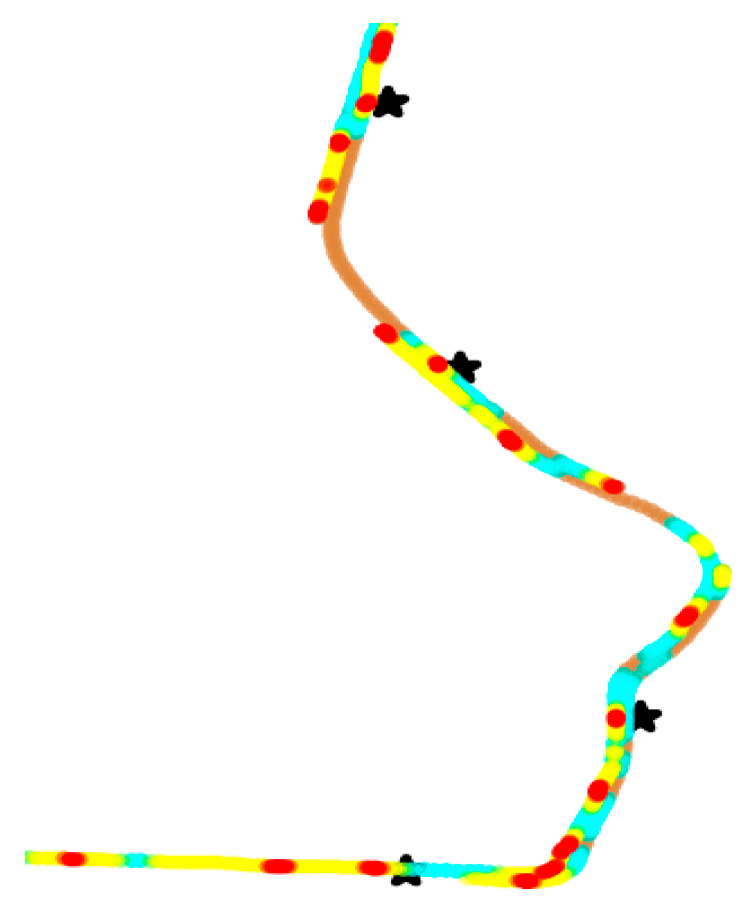
Detailed heat scatter plot of proper stopping at some bus stations (represented as “*”) with a low speed.

We calculated the bus speed within the 0–15 km/h interval, which is represented as the red dots in Figure 11. When drivers stop slowly, the red dots (of a low speed) coincides with the bus stations.

We also show the improper stopping at the bus stations where drivers accurately stop, as in the following cases.

**Figure 12:**
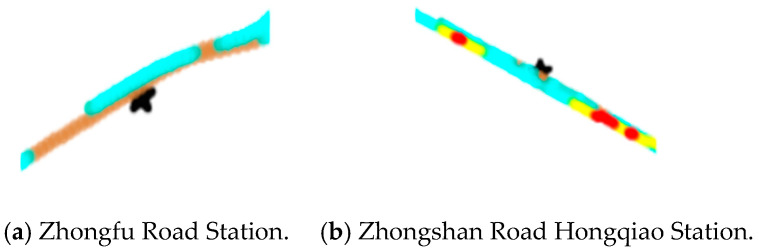
Detailed heat scatter plot of improper stopping at some bus stations (represented as “*”) of a high speed of the first trip in the morning.

From Figure 12 we suppose that the first bus avoids stopping at the Zhongfu Road Station and the Zhongshan Road Hongqiao Station. There is probably no passenger getting off the bus and no one waiting at the station in the early morning. Therefore, the drivers decide not to stop in the station area, which is a risky driving element.

(4) Average speed

Bus No. xxx has a one-way distance of 13.46 km. The first one-way journey takes 37 min, while it takes 61 min in the morning rush hours. The average one-way speed of the first shuttle is 21.8 km/h, and the average one-way speed of the morning-rush shuttle is 13.24 km/h.

(5) Fatigue driving

A comparison of the start and end times of Bus No. xxx on the same day reveals that a bus driver generally rests for 10–20 min at the end of each one-way trip and takes a 2 h lunch break at noon. The analysis shows that such a driving mode does not cause fatigue driving.

In all, the proposed GPS/BDS positioning and analysis indicate that the drivers of Bus No. xxx mainly follow actual road and traffic conditions, and exhibits some risky driving behaviors, such as speeding and braking urgently/stopping improperly. The monitoring of urban bus traffic behaviors can be vital to enable real-time public traffic management.

## 5. Conclusions

As one of the most important public transportation systems in cities, buses have a regularity that ordinary cars lack and an extensiveness that single fixed lines such as subways do not have. As such, they can be used as a carrier for studying the feedback and prediction of road condition information and infrastructure construction. The methods for obtaining an object’s trajectory, speed, acceleration, and other information from GPS/BDS positioning data for behavior analysis can be applied to bus drivers to ensure road safety. Some examples of such methods are an analysis and correction of the trainees’ driving behavior and implementation of behavioral safety regulations and evaluations for ride-hailing or taxi drivers. In this study, the GPS/BDS pseudorange differential positioning technology was adopted to greatly reduce the difficulty in the algorithm, which improved its feasibility and efficiency. The field results demonstrate that the tracking accuracy satisfies the requirement of bus behavior monitoring, which is an essential factor for modern urban planning, bus management, and traffic monitoring. In the future, efforts should be focused on improving the velocity and acceleration estimations with kinematic modes for various road conditions. Furthermore, we will identify bus drivers’ behavior patterns and apply evaluation methods for intelligent bus systems, i.e., traffic lights control, bus route planning, and proactive congestion management.

## Figures and Tables

**Table 1:** Parameters and definition of the bus vehicle terminal.

Parameter Name	Specific Parameters
Positioning system	GPS/BDS differential positioning
Center frequency	Using B1 frequency: 1561.098 Hz
Sensitivity	−133 dBm
Number of channels	12 independent BD2 B1 civilian code receiving channels
Single point positioning accuracy	PDOP ≤ 4; horizontal position ≤ 5 m; vertical position ≤ 8 m
Positioning time of receiver	Cold start time 30~45 s
Update rate	1 Hz
Timing accuracy	50 ns
Power supply	Rated 12 V
Power dissipation	≤3 W
Working temperature	−40 °C~+85 °C
Humidity	5% ~ 95%
Protection grade	IP65
Data packet loss rate	Less than 5%
Network communication	The communication is a 3G communication mode based on the TCP protocol for a long-distance connection
Delay setting	Data is automatically lost when the delay exceeds 3 s, and no longer transmitted to the server
Other	Support the remote configuration terminal instruction format

**Table 2:** Variable definition of various driving behaviors.

Risky Driving Behavior	Distance/m	Duration/s	Number of Times
Speeding	VS	VT	VN
Rapid acceleration	AAS	AAT	AAN
Sharp slowdown	ASS	AST	ASN

**Table 3:** DGNSS data format of the bus traces.

Data Notations	Byte String
Bus vehicle identification (ID)	32-byte string
Bus vehicle number (ZDBH)	15-byte string
GPS/BDS time (GNSSSJ)	YYYY-MM-DD HH24:MI:SS
Set up time (JLSJ)	YYYY-MM-DD HH24:MI:SS
Storage time (CCSJ)	YYYY-MM-DD HH24:MI:SS
Longitude (JD)	15-byte string
Latitude (WD)	15-byte string
Number of satellites (WXSL)	Two-digit integer

**Table 4:** Data instances of the acceleration values (m/s^2^).

Time (Start)	Time (End)	Acceleration (m/s^2^)
7:24:21	7:24:22	2.4012522902617603
7:26:49	7:26:50	1.8576729328204293
7:26:52	7:26:53	1.7403166489549329
7:28:28	7:28:29	1.7389263796751016
7:29:33	7:29:34	2.5543606480992884
7:29:37	7:29:38	2.0349395742103993
7:36:22	7:36:23	1.716697223852211
7:36:29	7:36:30	1.7642696573137318

## Data Availability

The data used to support the findings of this study are available from the corresponding author upon request.
